# A new aqua­manganese(II) oxalate phosphate, Mn(C_2_O_4_)Mn_3_(PO_4_)_2_(H_2_O)_2_

**DOI:** 10.1107/S160053680901201X

**Published:** 2009-04-02

**Authors:** Juan Zheng, Yuxia Shen, Tao Song, Aiyun Zhang

**Affiliations:** a Department of Physics and Chemistry, Henan Polytechnic University, Jiaozuo 454000, People’s Republic of China

## Abstract

The title salt, diaquatetra­manganese(II) oxalate bis[ortho­phos­phate(V)], Mn_4_(C_2_O_4_)(PO_4_)_2_(H_2_O)_2_, was synthesized hydro­thermally and displays a three-dimensional framework structure. The asymmetric unit consists of two different Mn^II^ centers, half of an oxalate anion, a phosphate group and a coordinated water mol­ecule. A crystallographic inversion center is located at the mid-point of the oxalate C—C bond. The distorted octa­hedral MnO_6_ and the tetra­gonal pyramidal MnO_5_ centers are linked through bridging oxalate and phosphate groups. The water mol­ecule also has a weaker bonding contact to the five-coordinate Mn atom, which consequently exhibits a distorted octa­hedral geometry and also bridges the independent Mn atoms. The water mol­ecule is a donor for intra- and inter­molecular O—H⋯O hydrogen bonds.

## Related literature

For the structure of HgC_2_O_4_ from synchrotron, X-ray and neutron powder diffraction data, see: Christensen *et al.* (1994 ▶). For a polymeric [Ni^II^(bpy)_3_]_*n*_^2+^ [Mn^II^(C_2_O_4_)_3_]_*n*_^2−^ oxalate-bridged network structure, see: Decurtins *et al.* (1994 ▶). For the structures of indium selenite–oxalate and indium oxalate, see: Cao *et al.* (2009 ▶). For lanthanide–oxalate coordination polymers, see: Zhang *et al.* (2009 ▶).

## Experimental

### 

#### Crystal data

Mn_4_(C_2_O_4_)(PO_4_)_2_(H_2_O)_2_
*M* *_r_* = 266.88
Monoclinic, 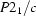

*a* = 10.2759 (2) Å
*b* = 6.5220 (1) Å
*c* = 10.0701 (1) Å
β = 116.926 (1)°
*V* = 601.73 (2) Å^3^
*Z* = 4
Mo *K*α radiation
μ = 4.45 mm^−1^
*T* = 296 K
0.21 × 0.19 × 0.17 mm

#### Data collection

Bruker APEXII CCD area-detector diffractometer
Absorption correction: multi-scan (*SADABS*; Bruker, 2007 ▶) *T*_min_ = 0.455, *T*_max_ = 0.519 (expected range = 0.412–0.470)
5971 measured reflections
1653 independent reflections
1467 reflections with *I* > 2σ(*I*)
*R*_int_ = 0.031

#### Refinement

*R*[*F* ^2^ > 2σ(*F* ^2^)] = 0.026
*wR*(*F* ^2^) = 0.065
*S* = 1.05
1653 reflections
106 parameters
3 restraints
H atoms treated by a mixture of independent and constrained refinement
Δρ_max_ = 0.47 e Å^−3^
Δρ_min_ = −0.55 e Å^−3^

### 

Data collection: *APEX2* (Bruker, 2007 ▶); cell refinement: *SAINT* (Bruker, 2007 ▶); data reduction: *SAINT*; program(s) used to solve structure: *SHELXS97* (Sheldrick, 2008 ▶); program(s) used to refine structure: *SHELXL97* (Sheldrick, 2008 ▶); molecular graphics: *SHELXTL* (Sheldrick, 2008 ▶); software used to prepare material for publication: *SHELXTL*.

## Supplementary Material

Crystal structure: contains datablocks I, global. DOI: 10.1107/S160053680901201X/si2161sup1.cif

Structure factors: contains datablocks I. DOI: 10.1107/S160053680901201X/si2161Isup2.hkl

Additional supplementary materials:  crystallographic information; 3D view; checkCIF report

## Figures and Tables

**Table 1:** Selected bond lengths (Å)

Mn1—O2^i^	2.1199 (15)
Mn1—O3^ii^	2.1407 (15)
Mn1—O1	2.1584 (15)
Mn1—O3^iii^	2.2219 (15)
Mn1—O5	2.2525 (15)
Mn1—O7*W*	2.2637 (17)
Mn2—O2^iv^	2.1145 (15)
Mn2—O4^ii^	2.1218 (15)
Mn2—O1	2.1220 (16)
Mn2—O6^v^	2.1809 (17)
Mn2—O5	2.2190 (16)
Mn2—O7*W*^vi^	2.5641 (14)

Symmetry codes: (i) 
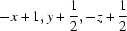
; (ii) 
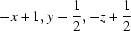
; (iii) 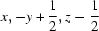; (iv) 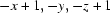; (v) 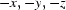; (vi) 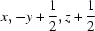.

**Table 2:** Hydrogen-bond geometry (Å, °)

*D*—H⋯*A*	*D*—H	H⋯*A*	*D*⋯*A*	*D*—H⋯*A*
O7*W*—H7*A*⋯O6	0.85 (2)	2.00 (2)	2.828 (3)	167 (3)
O7*W*—H7*B*⋯O4^vii^	0.86 (2)	1.95 (2)	2.787 (2)	167 (3)

Symmetry code: (vii) 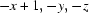.

**Fig. 1.**
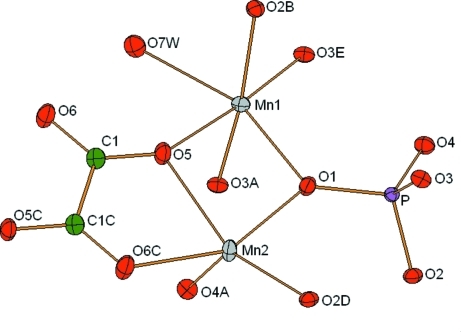
A section of the coordination geometry in the title polymer structure. Displacement ellipsoids are drawn at the 50% the probability level. Symmetrycodes: (A) = -x + 1, y - 1/2, -z + 1/2; (B) = -x + 1, y + 1/2, -z + 1/2; (C) -x, -y, -z; (D) = -x + 1, -y, -z + 1; (E) = x, -y + 1/2, z - 1/2;

**Fig. 2.**
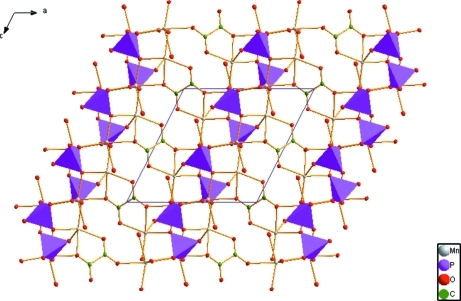
Packing diagram for Mn(C2O4)Mn3(PO4)2˙2H2O, viewed along the b axis.

**Fig. 3.**
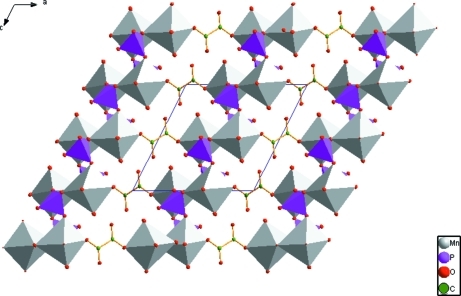
Packing diagram for Mn(C2O4)Mn3(PO4)2˙2H2O, viewed along the b axis, Mn2 complex cations are omitted for clarity.

## supplementary crystallographic information

### Comment

Over the past decades, the synthesis of new two and three dimensional inorganic materials has received great attention, due to their functional applications. Among the hybrid compounds are metal oxalates which exhibit vast diversity and unusual structural features. The oxalate anion displays various coordination modes when it is bound to metal cations. For example, the structures of HgC_2_O_4_(Christensen*et al., *1994), [In_2_(SeO_3_)_2_(C_2_O_4_)(H_2_O)_2_]˙2(H_2_O) (Cao *et al.*, 2009) and Nd(C_2_O_4_)(CH_3_COO)(H_2_O) (Zhang *et al.*, 2009) have been investigated in the past years. In this work, we designed and synthesized the title compound, MnC_2_O_4_Mn_3_(PO_4_)_2_˙2(H_2_O), which features a three-dimensional framework.

In the structure of the title compound, there are two Mn^II^ atoms, one phosphate, a half oxalate and one water per asymmetric unit (Fig. 1). Mn1 has a MnO_6_ octahedral coordination environment, but Mn2 is coordinated with five oxygen atoms (Fig. 2 and Fig. 3). The Mn—O oxalate distances (Table 1) are slightly longer than the Mn—O distances of 2.154 (2) Å and 2.166 (2) Å, observed in the polymeric anionic network structure [Ni^II^(bpy)_3_]^2+^_n_ [Mn^II^(C_2_O_4_)_3_]_n_^2-^ (Decurtins *et al.*, 1994). The distorted octahedral MnO_6_ and tetragonal pyramidal MnO_5_ centers are linked through bridging oxalate and phosphate groups (Fig. 3). The water molecule has also a weaker bonding contact to the five coordinate atom Mn2, which consequently exhibits a distorted octahedral geometry and bridges the independent atoms Mn1 and Mn2 as well. The water molecule is a donor for intra- and intermolecular O—H···O hydrogen bonds (Table 2).

### Experimental

Colorless block crystals were synthesized hydrothermally from a mixture of, H_3_BO_3_, H_2_C_2_O_4_, ethylenediamine, H_3_PO_4 _and water. In a typical synthesis, 0. 98 g MnCl_2_˙4H_2_O was dissolved in a mixture of 5 mL water, with 0.92 g H_3_BO_3_, 2 ml (85%) H_3_PO_4_ and 0.05 ml ethylenediamine at constant stirring. Finally, the mixture was kept in a 30 ml Teflon – lined steel autoclave at 443 K for 5 days. The autoclave was slowly cooled to room temperature. Colorless block crystals of the title compound were obtained.

### Refinement

The H atoms of the coordinated water molecule were refined with *U*_iso_(H) = 1.2*U*_eq_(O) and distance restraints d(O—H) of 0.85 (1) Å and d(H···H) of 1.33 (1) Å, respectively. The highest peak in the difference map is 0.47 e/Å^3^, and 0.75 Å from O4, and the minimum peak is -0.55 e/Å^3^, and 0.60 Å from Mn1.

### Crystal data

**Table:**

[Mn_4_(C_2_O_4_)(PO_4_)_2_(H_2_O)_2_]	*F*(000) = 516
*M**_r_* = 266.88	*D*_x_ = 2.946 Mg m^−^^3^
Monoclinic, *P*2_1_/*c*	Mo *K*α radiation, λ = 0.71073 Å
Hall symbol: -P 2ybc	Cell parameters from 2504 reflections
*a* = 10.2759 (2) Å	θ = 2.2–29.9°
*b* = 6.5220 (1) Å	µ = 4.45 mm^−^^1^
*c* = 10.0701 (1) Å	*T* = 296 K
β = 116.926 (1)°	Block, colourless
*V* = 601.73 (2) Å^3^	0.21 × 0.19 × 0.17 mm
*Z* = 4	

### Data collection

**Table:**

Bruker APEXII CCD area-detector diffractometer	1653 independent reflections
Radiation source: fine-focus sealed tube	1467 reflections with *I* > 2σ(*I*)
graphite	*R*_int_ = 0.031
φ and ω scans	θ_max_ = 29.9°, θ_min_ = 2.2°
Absorption correction: multi-scan (*SADABS*; Bruker, 2007)	*h* = −14→11
*T*_min_ = 0.455, *T*_max_ = 0.519	*k* = −9→8
5971 measured reflections	*l* = −13→13

### Refinement

**Table:**

Refinement on *F*^2^	Primary atom site location: structure-invariant direct methods
Least-squares matrix: full	Secondary atom site location: difference Fourier map
*R*[*F*^2^ > 2σ(*F*^2^)] = 0.026	Hydrogen site location: inferred from neighbouring sites
*wR*(*F*^2^) = 0.065	H atoms treated by a mixture of independent and constrained refinement
*S* = 1.05	*w* = 1/[σ^2^(*F*_o_^2^) + (0.0352*P*)^2^ + 0.1904*P*] where *P* = (*F*_o_^2^ + 2*F*_c_^2^)/3
1653 reflections	(Δ/σ)_max_ < 0.001
106 parameters	Δρ_max_ = 0.47 e Å^−^^3^
3 restraints	Δρ_min_ = −0.55 e Å^−^^3^

### Special details

**Table:**

Geometry. All e.s.d.'s (except the e.s.d. in the dihedral angle between two l.s. planes) are estimated using the full covariance matrix. The cell e.s.d.'s are taken into account individually in the estimation of e.s.d.'s in distances, angles and torsion angles; correlations between e.s.d.'s in cell parameters are only used when they are defined by crystal symmetry. An approximate (isotropic) treatment of cell e.s.d.'s is used for estimating e.s.d.'s involving l.s. planes.
Refinement. Refinement of *F*^2^ against ALL reflections. The weighted *R*-factor *wR* and goodness of fit *S* are based on *F*^2^, conventional *R*-factors *R* are based on *F*, with *F* set to zero for negative *F*^2^. The threshold expression of *F*^2^ > σ(*F*^2^) is used only for calculating *R*-factors(gt) *etc*. and is not relevant to the choice of reflections for refinement. *R*-factors based on *F*^2^ are statistically about twice as large as those based on *F*, and *R*- factors based on ALL data will be even larger.

### Fractional atomic coordinates and isotropic or equivalent isotropic displacement parameters (Å^2^)

**Table:**

	*x*	*y*	*z*	*U*_iso_*/*U*_eq_	
Mn1	0.38352 (4)	0.14623 (5)	0.02672 (4)	0.01004 (10)	
Mn2	0.26622 (4)	−0.02679 (6)	0.26753 (4)	0.01198 (10)	
P1	0.60110 (6)	0.15130 (8)	0.39476 (6)	0.00740 (13)	
O1	0.45405 (17)	0.0984 (2)	0.26145 (16)	0.0123 (3)	
O2	0.65691 (17)	−0.0342 (2)	0.50294 (16)	0.0110 (3)	
O3	0.57805 (17)	0.3259 (2)	0.48610 (17)	0.0112 (3)	
O4	0.71121 (17)	0.2029 (2)	0.33744 (17)	0.0124 (3)	
O5	0.17132 (17)	0.0906 (3)	0.03574 (17)	0.0148 (3)	
O6	−0.03134 (18)	0.0699 (3)	−0.18037 (17)	0.0174 (4)	
O7W	0.22135 (19)	0.1426 (2)	−0.21781 (18)	0.0157 (4)	
H7A	0.1384 (17)	0.118 (4)	−0.222 (3)	0.019*	
H7B	0.231 (3)	0.043 (3)	−0.268 (3)	0.019*	
C1	0.0400 (2)	0.0465 (3)	−0.0428 (2)	0.0122 (4)	

### Atomic displacement parameters (Å^2^)

**Table:**

	*U*^11^	*U*^22^	*U*^33^	*U*^12^	*U*^13^	*U*^23^
Mn1	0.01109 (18)	0.00852 (19)	0.01143 (17)	0.00060 (12)	0.00591 (14)	0.00116 (11)
Mn2	0.00891 (18)	0.0177 (2)	0.00885 (17)	−0.00151 (13)	0.00360 (14)	−0.00260 (12)
P1	0.0080 (3)	0.0070 (3)	0.0073 (2)	−0.00008 (19)	0.0035 (2)	−0.00015 (18)
O1	0.0098 (8)	0.0162 (8)	0.0092 (7)	−0.0004 (6)	0.0027 (6)	0.0008 (6)
O2	0.0140 (8)	0.0081 (8)	0.0102 (7)	0.0009 (6)	0.0050 (6)	0.0013 (5)
O3	0.0140 (8)	0.0099 (8)	0.0126 (7)	−0.0005 (6)	0.0084 (7)	−0.0016 (6)
O4	0.0129 (8)	0.0130 (8)	0.0133 (7)	−0.0006 (6)	0.0078 (6)	0.0011 (6)
O5	0.0088 (8)	0.0214 (9)	0.0121 (7)	−0.0022 (7)	0.0031 (6)	0.0005 (6)
O6	0.0105 (8)	0.0299 (10)	0.0111 (7)	−0.0017 (7)	0.0041 (7)	0.0022 (7)
O7W	0.0160 (9)	0.0179 (9)	0.0156 (8)	−0.0021 (7)	0.0091 (7)	−0.0014 (6)
C1	0.0106 (10)	0.0137 (11)	0.0134 (10)	0.0008 (8)	0.0064 (9)	−0.0014 (8)

### Geometric parameters (Å, °)

**Table:**

Mn1—O2^i^	2.1199 (15)	P1—O1	1.5386 (16)
Mn1—O3^ii^	2.1407 (15)	P1—O3	1.5481 (15)
Mn1—O1	2.1584 (15)	P1—O2	1.5534 (15)
Mn1—O3^iii^	2.2219 (15)	O2—Mn2^iv^	2.1145 (15)
Mn1—O5	2.2525 (15)	O2—Mn1^ii^	2.1199 (15)
Mn1—O7W	2.2637 (17)	O3—Mn1^i^	2.1407 (15)
Mn2—O2^iv^	2.1145 (15)	O3—Mn1^vi^	2.2219 (15)
Mn2—O4^ii^	2.1218 (15)	O4—Mn2^i^	2.1218 (15)
Mn2—O1	2.1220 (16)	O5—C1	1.250 (3)
Mn2—O6^v^	2.1809 (17)	O6—C1	1.249 (3)
Mn2—O5	2.2190 (16)	O7W—H7A	0.85 (2)
Mn2—O7W^vi^	2.5641 (14)	O7W—H7B	0.86 (2)
P1—O4	1.5225 (15)	C1—C1^v^	1.558 (4)
			
O2^i^—Mn1—O3^ii^	168.73 (6)	O4—P1—O1	108.88 (8)
O2^i^—Mn1—O1	104.10 (6)	O4—P1—O3	113.75 (9)
O3^ii^—Mn1—O1	86.86 (6)	O1—P1—O3	109.23 (9)
O2^i^—Mn1—O3^iii^	91.66 (6)	O4—P1—O2	109.64 (9)
O3^ii^—Mn1—O3^iii^	82.11 (6)	O1—P1—O2	110.00 (9)
O1—Mn1—O3^iii^	109.24 (6)	O3—P1—O2	105.28 (8)
O2^i^—Mn1—O5	91.86 (6)	P1—O1—Mn2	127.44 (9)
O3^ii^—Mn1—O5	93.07 (6)	P1—O1—Mn1	129.28 (9)
O1—Mn1—O5	77.51 (6)	Mn2—O1—Mn1	103.21 (7)
O3^iii^—Mn1—O5	171.35 (6)	P1—O2—Mn2^iv^	117.17 (8)
O2^i^—Mn1—O7W	81.74 (6)	P1—O2—Mn1^ii^	132.87 (9)
O3^ii^—Mn1—O7W	89.39 (6)	Mn2^iv^—O2—Mn1^ii^	106.93 (6)
O1—Mn1—O7W	155.02 (6)	P1—O3—Mn1^i^	126.88 (9)
O3^iii^—Mn1—O7W	94.65 (6)	P1—O3—Mn1^vi^	124.19 (9)
O5—Mn1—O7W	78.05 (6)	Mn1^i^—O3—Mn1^vi^	97.89 (6)
O4^ii^—Mn2—O7W^vi^	154.66 (7)	P1—O4—Mn2^i^	129.80 (9)
O2^iv^—Mn2—O4^ii^	129.04 (6)	C1—O5—Mn2	114.85 (14)
O2^iv^—Mn2—O1	93.71 (6)	C1—O5—Mn1	143.03 (14)
O4^ii^—Mn2—O1	89.96 (6)	Mn2—O5—Mn1	97.22 (6)
O2^iv^—Mn2—O6^v^	105.17 (6)	C1—O6—Mn2^v^	114.67 (14)
O4^ii^—Mn2—O6^v^	92.47 (6)	Mn1—O7W—H7A	106.7 (18)
O1—Mn2—O6^v^	153.30 (6)	Mn1—O7W—H7B	115.2 (18)
O2^iv^—Mn2—O5	148.61 (6)	H7A—O7W—H7B	101.8 (13)
O4^ii^—Mn2—O5	81.83 (6)	O6—C1—O5	126.6 (2)
O1—Mn2—O5	78.99 (6)	O6—C1—C1^v^	118.0 (2)
O6^v^—Mn2—O5	75.05 (6)	O5—C1—C1^v^	115.4 (2)

Symmetry codes: (i) −*x*+1, *y*+1/2, −*z*+1/2; (ii) −*x*+1, *y*−1/2, −*z*+1/2; (iii) *x*, −*y*+1/2, *z*−1/2; (iv) −*x*+1, −*y*, −*z*+1; (v) −*x*, −*y*, −*z*; (vi) *x*, −*y*+1/2, *z*+1/2.

### Hydrogen-bond geometry (Å, °)

**Table:**

*D*—H···*A*	*D*—H	H···*A*	*D*···*A*	*D*—H···*A*
O7W—H7A···O6	0.85 (2)	2.00 (2)	2.828 (3)	167 (3)
O7W—H7B···O4^vii^	0.86 (2)	1.95 (2)	2.787 (2)	167 (3)

Symmetry codes: (vii) −*x*+1, −*y*, −*z*.
